# Chemical Composition and Antibacterial Activities of Eight Plant Essential Oils from Morocco against *Escherichia coli* Strains Isolated from Different Turkey Organs

**DOI:** 10.1155/2021/6685800

**Published:** 2021-03-15

**Authors:** Hassna Jaber, Asmaa Oubihi, Imane Ouryemchi, Rachid Boulamtat, Ali Oubayoucef, Brahim Bourkhiss, Mohammed Ouhssine

**Affiliations:** ^1^Laboratory of Natural Resources and Sustainable Development, Department of Biology, Faculty of Sciences, Ibn Tofail University, Kenitra, Morocco; ^2^Laboratory of Biology and Health, Department of Biology, Faculty of Sciences Ibn Tofail University, Kenitra, Morocco; ^3^Laboratory of Biochemistry and Immunology, Department of Biology, Faculty of Sciences Mohamed V University, Rabat, Morocco

## Abstract

The aim of the present study was to determine the chemical composition of eight plant essential oils and evaluate their antibacterial activity against *Escherichia coli* strains isolated from different turkey organs. The essential oils were extracted by hydrodistillation and analyzed using gas chromatography-mass spectroscopy. All essential oil yielded high in a range between 2.2 and 3.12%. Gas chromatography-mass spectroscopy (GC-MS) revealed that the major constituents of *Thymus vulgaris, Ocimum basilicum, Artemisia herba-alba,* and *Syzygium aromaticum* oils were thymol (41.39%), linalool (37.16%), camphor (63.69%), and eugenol (80.83%), respectively. Results of the *E. coli* sensitivity evaluated by the standard antimicrobial sensitivity method varied depending on the organ of isolation. Similarly, the essential oils antimicrobial activity determined by the disc diffusion method varied all along within the organs of isolation. *T. vulgaris* essential oil showed the highest effective antibacterial activity against *E. coli* isolated from the throat with an inhibition zone diameter value of up to 23.33 mm. However, all the essential oils showed antibacterial activity and the MIC and MBC values were in the range of 1/3000 to 1/100 (v/v) and the ratios MBC/MIC were equal to 1. In conclusion, this study showed that the essential oils could be promising alternatives to overcome *E. coli* multiresistance in turkey.

## 1. Introduction

Poultry is the second most widely eaten meat worldwide accounting for over 35% of the world's consumed meat [1]. In fact, to match the increasing demand of the consumers, all along with their requirements in terms of quality and taste, the use of antibiotics as growth promoters has been imposed in the poultry industry. Nowadays, it has become a widespread practice in poultry breeding. The main roles of antibiotics are to control infectious animal diseases, increase their weight, and improve their performance [2]. Avian colibacillosis is an example of a serious poultry disease caused by avian pathogenic *Escherichia coli* strains, and it could be spread by fecal-oral cycle, contaminated dust, and hatched egg residues [3]. Several studies have shown that poultry is a very important source of contamination for humans and a large number of potential human pathogenic bacteria species are found in poultry meat, e.g., *Enterococcus faecalis*, *Enterococcus faecium*, *Staphylococcus aureus* [4], and *Salmonella enterica* [5]. A recent meta-analysis of European published surveys between 2000 and 2017 revealed that *S. aureus* is the main pathogen detected in poultry meat, followed by *Campylobacter* spp. [6]. Contaminated food with pathogenic microbes causes a variety of symptoms known as food-borne diseases and these symptoms vary from mild to severe and sometimes fatal [7]. Meat handlers and consumers are at risk of food-borne infections from *E. coli* that are resistant to most antibiotics in use [8]. In the last few years, the overuse of antibiotics in poultry breeding and other industries has led to the appearance of antibiotic residues and antibiotic resistant bacteria which has numerous threats to the environment and the consumers' health [9]. Antibiotic residues alter the microbial population and create a selection pressure towards resistant strains in the environment, and the consumption of antibiotic-containing meat products alters the human microbiome with threats of emerged resistant strains in the human body or altering the benefits of the symbiotic bacteria [10]. Furthermore, some antibiotic residues or their metabolic derivatives may lead to some health effects on humans; even though the direct mechanism is not well understood, a couple of studies suggest that antibiotic residues are associated with obesity, carcinogenicity, reproductive effects, and teratogenicity [11]. Apata [12] reviewed the antibiotic resistance in poultry and provided multiple examples like the use of fluoroquinolones antibiotic and the emergence of resistant *Campylobacter* in 10% of human *Campylobacter* diseases found in the area of the antibiotic introduction.

Banning the use of antibiotics as growth promoters remains possible and presents a major challenge for poultry meat production, hence the need to find new methods or alternatives in order to overcome this problem [13, 14]. Natural substances are in great demand in the field of bio-farming also known as organic farming systems, and thus plant derivative products are gaining increased attention at different levels in the chain industry, mainly as immune-modulators [15]. Plant derivatives not only have the potential of antibiotic alternatives in terms of growth promoters and disease control [16] but also have the potential to increase meat and egg quality [17, 18]. Furthermore, encapsulated essential oils have been shown to be effective to control necrotic enteritis in broiler chickens [19]. Moreover, EOs have been tested as natural food additives for protection and flavouring purposes [20]. Overall, Adaszyńska-Skwirzyńska and Szczerbińska [21] have shown strong evidence of essential oils' positive effects on the growth, health, and quality of poultry meat. Many essential oils and plant extracts have been widely used in traditional medicine and nowadays are gaining increased attention in modern medicine, cosmetic practices, and agricultural and food industry [22, 23]. However, their potential application in animal production, especially poultry, remains largely unexploited.

The objective of this work was to search for new alternatives to antibiotics used in the poultry industry. And therefore, eight EOs of Moroccan aromatic plants were tested regarding their antimicrobial activity against *E. coli* isolated from different raw turkey organs. The choice of these plants was made after a preliminary study that included more than 20 plants that are popular in Morocco and used for various practices [24, 25].

## 2. Materials and Methods

### 2.1. Plant Material

The plants used in this study were as follows: *Thymus vulgaris*, *Artemisia herba-alba*, *Ocimum basilicum*, *Syzygium aromaticum*, *Eucalyptus globulus*, *Mentha pulegium*, *Mentha spicata*, and *Rosmarinus officinalis*. Samples were collected from various areas of Morocco, brought to our laboratory, and prepared for the EOs extraction.

### 2.2. Microorganism

The study was carried out on 168 samples from different parts of the turkey meat: breast (*n* = 24), liver (*n* = 24), thigh (*n* = 24), upper thigh (*n* = 24), throat (*n* = 24), garlic (*n* = 24), and skin (*n* = 24). Each sample was collected in the morning and placed in a sterile stomacher bag and then transported to the laboratory in a cooler (4°C). Upon arrival, the samples were immediately analyzed in three repetitions. A portion of 25 g of each sample was cut aseptically and then crushed carefully using a chopper Ultra-Turrax and then added in an Erlenmeyer flask containing 225 ml of sterile physiological water, from which we have prepared a tenfold dilution series ranging up to 10^−6^ (NM 08.0.117, 2007). *E. coli* isolation was carried out on MAC Conckey (CM 105 Oxoid, Germany) medium by cooling 1 ml of each dilution on the plate and cultured for 24 h at 37°C (NM 08.0.127, 2012). Colonies of big size (>0.5 mm diameter) and surrounded with a dark pink area were considered to be *E. coli* colonies. The identification of isolated *E. coli* strains was done on a total of 168 analyzed samples, and 82 isolates of *E. coli* were identified. The identification was carried out with a classical gallery and the confirmation was made by the gallery API 20E identification system (bioMerieux, France).

### 2.3. Antibiotics

Three antibiotics were selected as positive checks based on the resistance-sensibility profiles of the studied *E. coli* collection [26]. And they were as follows: streptomycin (ST) 300 *μ*g, colistin (CT) 50 *μ*g, and gentamicin (GT) 15 *μ*g. The antibiotics were kindly provided by the National Hygiene Institute in Rabat, Morocco.

### 2.4. EOs Extraction

Plant samples were powdered and macerated in distilled water for 24 h in a ratio of 1/10 (g/ml). Then, the EOs were obtained by 3 h hydrodistillation using the Clevenger-type system. EOs were collected, determined for extraction yield, and stored in screw-capped glass vials at 4°C until use.

### 2.5. GC-MS Analysis

Chromatographic analyses of eight essential oils were carried out in CNRST institute, Morocco: 1 *μ*l of the liquid of each sample was dissolved in an appropriate volume of chloroform. The profile of volatile compounds was characterized by gas chromatography (GC) (Hewlett Packard 5890 II) coupled to mass spectrometry (MS) (Hewlett Packard 5972 MSD operating in the EI mode at 70 eV) equipped with a VB-5 capillary column (methylpolysiloxane 5% phenyl) (30 m × 0.25 mm) with a film thickness of 0.25 *μ*m, an FID detector set at 300°C and supplied by a mixture of H_2_/Air-gas, and a split-splitless injector set at 220°C. 1 *μ*l of the soluble extract was injected into the column by 1 : 50 split mode using helium as carrier gas at 1.4 ml min^−1^. The temperature was programmed from 40 to 300°C at 4°C/min and a plateau of 5 minutes at the final temperature. The identification of the compounds was based on the comparison of their relative retention indexes and mass spectra with those of NIST 98 and Wiley 275 library data. The percentages relative of the compounds were obtained electronically from area percent data.

### 2.6. Antibiotics Sensitivity

In this study, 3 antibiotics were chosen according to their natural spectrum, their authorization in poultry production, and their wide use in the treatment of avian colibacillosis in Morocco [27]. The antibiotics sensitivity was carried out following the standard antimicrobial sensitivity method [28] using Mueller-Hinton medium (CM 0337 Oxoid, UK), and antibiotic discs (Thermo Scientific™ Oxoid™, Sweden). The bacterial suspension was prepared from viable colonies for 24 to 48 h, well homogenized in 8.5% NaCl solution. After inoculum preparation, flood inoculation was performed on the entire surface of the nutrient Mueller-Hinton agar medium (CM 0337 Oxoid, England). From the application of the discs impregnated with specific antibiotics to be tested, the antibiotics diffuse uniformly. After incubation, at 36 ± 1°C, for 24 h, the discs were surrounded by circular zones of inhibition. The strains were categorized as sensitive (S), intermediate (I), or resistant (R) according to the critical diameters provided by the Antibiogram Committee of the French Microbiology Society [29, 30]. The *E. coli* reference strain ATCC® 25922™ was used as a control strain (Table 1).

### 2.7. EOs Antibacterial Tests

#### 2.7.1. Antibacterial Activity Screening

The antibacterial activity of the EOs was evaluated by the disk diffusion method which is recognized as reliable and reproducible; it is mainly used in a preliminary stage of the in-depth studies because it gives access to qualitative results [31, 32]. The method was used with slight modifications [33]. Briefly, it is based on depositing a sterile disk, pre-soaked in EOs, on a bacterial carpet at the beginning of the growth and measuring the zone where the bacteria could not develop: the inhibition zone diameter value, which reflects the antibacterial activity of the EOs.

A bacterial suspension of density equivalent to 0.5 McFarland standards (10^8^ CFU/ml) was prepared and then diluted to 1/100. 15 ml of Mueller-Hinton agar medium was poured on per Petri dish. 2 ml of the inoculum was deposited on each Petri dish. After 5 minutes of impregnation, the excess inoculum was removed. On the surface of each Petri dish, the sterile filter paper discs of 6 mm (Whatman paper No. 1, Oxoid) were impregnated with 15 *μ*l EOs and placed on the inoculated Petri dishes. After having remained at 4°C for 2 h, the Petri dishes were incubated at 37°C for 24 h [34]. After incubation, the inhibition zone diameter was measured in millimeters, including the disk's surface. Each test was performed three times in three successive experiments.

#### 2.7.2. Minimum Inhibitory Concentration (MIC)

The minimum inhibitory concentration (MIC) is defined as the smallest concentration of the product for which no bacterial growth is visible compared to the control, after an incubation time at 37°C for 24 h [35].

The minimum inhibitory concentrations (MIC) of essential oils were determined according to the method reported by Remmal et al. [36] and Satrani et al. [37]. Due to the non-miscibility of the essential oils with water and therefore with the culture medium, the emulsification was carried out using a 0.2% agar solution in order to facilitate the germ/compound contact.

Dilutions were prepared at 1/10^th^, 1/25^th^, 1/50^th^, 1/100^th^, 1/200^th^, 1/300^th^, and 1/500^th^ in this agar solution. 1 ml of each of the dilutions was added so as to obtain the final concentrations of 1/100, 1/250, 1/500, 1/1000, 1/2000, 1/3000, and 1/5000 (v/v). The tubes were then shaken well before pouring them into Petri dishes. Controls, containing the culture medium and the 0.2% agar solution alone, were also prepared. The experiment was carried out in triplicate and the MIC was determined by the lowest EO concentration that totally inhibits bacterial growth.

#### 2.7.3. Minimal Bactericidal Concentration (MBC)

MBC was used to reconfirm the results of MIC by determining the number of surviving organisms through observing the growth of the bacteria. The minimum bactericidal concentrations (MBCs) were done on the nutrient agar poured into Petri dishes, which were streaked with the contents of the dishes having a concentration ≥ MIC in the previous dilution series [38]. The MBCs determined from the test dishes showing no visible growth during the MIC experiments were inoculated by using the plates which had fresh Mueller–Hinton agar. The inoculated dishes were incubated for 24 h at 37°C. The experiment was carried out in triplicate and the concentration at which no growth was observed visibly from the plates was recorded as the MBC of the EOs that can kill more than 99.9% of the initial bacterial inoculum [39].

#### 2.7.4. Antibacterial Effect Interpretation

The ratio MBC/MIC was calculated to determine the efficacy and the bactericidal/bacteriostatic effect on the bacterial growth of *E. coli* [39]. If the MBC/MIC is ≤4, then the effect is bactericidal, while if MBC/MIC is >4, then the effect is bacteriostatic [40].

### 2.8. Statistical Analysis

All experimental data were analyzed using analysis of variance (ANOVA), at a significance level of *P* < 0.01. The data are expressed as percentages values for each measurement.

## 3. Results

### 3.1. Extraction Yield

The plant species tested in this study indicate moderate yields of EOs obtained by distillation, which varies between the eight plants from 2.2 to 3.12% (Table 2).

### 3.2. Chemical Constituents of Essential Oils

The chemical composition of the eight EOs that was analyzed by GC-MS is given in Table 3 all together with the percentage of each molecule and the total cumulative areas of all the constituents. Results show that the major compounds of *T. vulgaris*, *O. basilicum*, *A. herba alba*, *M. spicata*, *M. pulegium*, *R. officinalis*, *E. globulus*, and *S. aromaticum* were thymol, linalool, camphor, carvone, neo-menthol, eucalyptol (1.8 cineole), and eugenol, respectively.

### 3.3. Antibiotics Sensitivity

Results of the antibiotics sensitivity are presented in Table 4. All isolates from the seven organs were sensitive to gentamicin. Isolates from the thigh and breast were resistant to both streptomycin and colistin at the same time, while isolates from the upper thigh and liver were resistant to streptomycin and colistin, respectively. However, colistin and streptomycin had either medium or high activity against the rest of the isolates.

### 3.4. EOs Antimicrobial Activity

The screening for antimicrobial activity was done by the disc diffusion method and results are presented in Table 5. All the EOs exhibited antibacterial activity and could be grouped into 3 groups: (a) *E. globulus, M. pulegium, M. spicata*, and *R. officinalis* with a zone of inhibition around <10 mm; (b) *A. herba-alba, O. basilicum,* and *S. aromaticum* with a zone of inhibition between 10 and 20 mm; and (c) *T. vulgaris* which was the most effective EO against almost all the isolates with a zone of inhibition around 20 mm.

### 3.5. Measurement of the Antibacterial Activity of EOs

The minimum inhibitory concentration MIC and the minimum bactericidal concentration MBC and their ratio MIC/MBC were done to measure the antibacterial activity of the EOs and to determine whether they act as bacteriostatic or bactericidal agents and the results are presented in Table 6.

## 4. Discussion

Antibiotics resistance has been a rising issue during the past couple of decades, and the poultry industry contributed to the problem mainly by excessive use of these antibiotics [41]. Indeed, our results showed streptomycin and/or colistin resistant *E. coli* strains from different turkey organs. Antibiotic multiresistant bacteria are a serious threat to both animal and humans' health and difficult to cure as they resist commonly used antibiotics [42]. However, our experiments showed that gentamicin was effective against all the isolates. Previous studies have reported the effectiveness of gentamicin against *E. coli* strains with a sensitivity reaching up to 90% [43]. Filali et al. [44] reported a 100% efficacity of colistin against *E. coli* strains isolated from chickens with colisepticaemia. Furthermore, other pathogenic bacteria isolated from poultry meat such as *Salmonella* [45], *Listeria* [46], and *Campylobacter* [47] have also developed antibiotic resistance towards one or more antibiotics. Chaiba et al. [48] spotted Methicillin-resistant *S. aureus* isolated from poultry farmers and chicken slaughters which stresses even more the fact that poultry could be a reservoir for antibiotic resistant microbes that could easily infect humans especially those with direct contact like farmers.

In this context, alternatives to antibiotics are an urgent need to reduce their use in the poultry industry, i.e., plant essential oils. These plant derivatives are rich in secondary metabolites that could play a variety of functions in plant defense mechanisms towards herbivores and pathogenic bacteria and fungi. Plant secondary metabolites can act in a selective or non-selective way to trigger cell death. Furthermore, EOs are rich in hydrophobic molecules that could simply disrupt cell membrane fluidity and/or functionality [49]. Numerous studies were published about the use of EOs as antimicrobial agents, and especially against food-borne pathogens and their potential use in the food industry [50]. Regarding the yields, *M. pulegium* EO was the highest yield found in our study (3.12%) which was slightly similar to 3.30% found by Hmiri et al. [51]. The second yield in our study was obtained from *E. globulus* (3%); this value is close to 2.5% found by Farah et al. [52]. *T. vulgaris* and *A. herba-alba* yielded 2.2% and 2.84% EO in our work which is significantly higher than reported yields from eastern Morocco (1%) [53] and Tunisia (0.65%) [54], respectively. Regarding the composition, we found that the major components of *T. vulgaris* were thymol (41.39%) and camphor (38.5%), whereas Cheurfa et al. [55] found thymol (27.43%) and carvacrol (34.62%). Concerning *O. basilicum*, linalool (37.2%), methyl chavicol (14.3%), and limonene (13.2%) represent the major components of this oil. This finding is confirmed by Hanif et al. [56]. In the essential oil of *A. herba-alba*, the main components are camphor (32.82%), chrysanthenone (24.1%), and davanone (15.1%); this result agrees with that reported by Kadri et al. [57]. Regarding *S. aromaticum,* the main compound was eugenol (80.83%) which is in agreement with the finding of Moemenbellah‐Fard et al. [58]. The variation in EOs composition is linked to several factors such as the extraction methods, plant parts and genotypes, geographical origin and environment conditions, the harvesting period, the degree of drying, and drying conditions [59].

Our finding shows that antimicrobial activity of *T. vulgaris* was the most effective EO against almost all the isolates with a zone of inhibition around 20 mm, and this plant showed an important sensitivity against the *E. coli* strains in comparison with antibiotics tested and recorded the highest value of ID = 23.33 mm. This result is similar to those reported by Lambert and al. [60] and Al-Shuneigat et al. [61]. This bioactivity is in relation to its high percentage of carvacrol and thymol; these two phenolic compounds are known for their antimicrobial properties [62, 63]. The inhibition zones of *A. herba-alba, O. basilicum*, and *S. aromaticum* EOs ranged between 10 and 20 mm. Those three EOs present a powerful antibacterial effect similar to the tested antibiotics (gentamicin, streptomycin, and colistin) on *E. coli* strains, strongly higher than those reported by Zouari et al. [64], Shirazi et al. [65], and Oussalah et al. [66]. These activities could be attributed to chrysanthenone, camphor, *α*-terpin-7-Al, and trans-*β*-terpineol for *A. herba-alba* [57, 67], and linalool and methyl chavicol for *O. basilicum* [9, 68], and eugenol for *S. aromaticum* [69].

Our results show that the concentration to inhibit the development of germs (MIC and MBC) for *T. vulgaris* was 1/3000 (v/v); in the same way, other studies showed that *T. vulgaris* needs 1.33 mg/ml [53]. The MIC and MBC of *A. herba-alba* and *O. basilicum* were 1/2000 (v/v). Other authors reported that the concentrations were for 0.4% *O. basilicum* [70], and 1/500 (v/v) for *A. herba-alba* [71], while the concentration we found for *S. aromaticum* (1/1000 (v/v)) was similar to that reported by Leuschner and Lelsch [72]. This discrepancy is related to different methods used for the determination of MIC. For all the tested EOs, the MICs were equal to the MIBs and the MIC/MIB ratios were equal to 1 and thus the activities were bactericidal. Furthermore, the MIC did not vary across the different isolates for all the EOs and thus, the organs of isolation had no effect on the sensitivity of the isolates towards the EOs. To clarify, there is a divergence in the units of measurement used for values of MIC and MBC (%, *μ*l/ml, mg/ml, *μ*g/ml). In other studies, the MICs and MBCs are expressed also in v/v or w/v (m: weight, v: v). Consequently, the comparison data on MIC and MBC recorded for EOs tested is difficult.

On the other hand, the microbes used in our study were isolated from turkey organs and their antibiotic sensitivity was analyzed and as shown earlier; some of them developed antibiotic resistance towards streptomycin and/or colistin. Our results reflect more the efficiency of the tested EOs against *E. coli* strains isolated from the turkey organs. This activity is related to the different chemotypes of essential oils such as thymol, camphor, linalool, and eugenol. Those molecules are well known by their high antimicrobial activity against pathogenic bacteria (*E. coli* and *Salmonella* spp.) [60, 67, 71, 73].

This antibacterial activity acts by perturbating the cell membranes permeability, cell balance, and inhibiting the membrane-bound ATPase activity, and as a result, reducing the growth rate of pathogens and disrupting substance influx and even cell death [74, 75]. Several studies suggest that Gram-negative bacteria are more tolerant to the essential oil actions because of the hydrophilic membrane constituents [76, 77], in detail, a disruption of the membrane integrity and ion transport processes, by rapid dissipation of H^+^ and K^+^ ion gradients which reduce ATP synthesis and the increased hydrolysis [78].

Furthermore, EOs activity could be easily enhanced either by using a mixture of them or mixing them with other antibiotic agents [78, 79]. Such a strategy allows the possibility of using very low doses (below the MIC) of EOs without reducing the efficiency and also diversifying the mechanism of action and thus decreasing the odds for resistance development.

## 5. Conclusions

EOs are generally recognized as safe for use in the food and feed industry and the accumulation of their components in the body is unlikely due to their rapid elimination, despite the fact that the understanding of EOs mode of action is a prerequisite for their regular application in animal production. The present study clearly demonstrates the potential use of the studied EOs as antibiotics alternatives in the poultry industry, in particular, those extracted from *T. vulgaris*, *A. Herba alba, O. basilicum*, and *S. aromaticum*. Our results were obtained against *E. coli* isolated from different turkey organs. Even though the isolates had different antibiotics' sensitivity, they had the same reaction towards each tested EO and the organ of isolation seemed to have no effects on these interactions. Further studies are highly encouraged to confirm these results in in vivo scenarios incorporating the coast-effect benefits for more insightful results. The beneficial effects of EOs dietary supplementation should be taken into consideration to justify the additional cost of their application. Despite their strong antimicrobial action, the use of EOs in farms is limited by their poor water solubility. This characteristic makes it necessary to convey them with suitable surfactants or through biotechnological processes.

## Figures and Tables

**Table 1:** Phenotypic characterization of the antibiotic resistance of *Escherichia coli* ATCC® 25922™ strains according to the diameter of inhibition (mm) of three antibiotics [30].

Antibiotics	Dose (*μ*g)	Resistant (mm)	Intermediate (mm)	Sensitive (mm)
Colistin	50	<15	—	≥18
Gentamicin	15	<16	16–17	≥18
Streptomycin	300	<13	13–14	≥15

**Table 2:** The essential oil yields.

Plant species	EOs yield (%)
*T. vulgaris*	2.2
*A. herba-alba*	2.84
*O. basilicum*	2.1
*S. aromaticum*	2.34
*E. globulus*	3
*M. pulegium*	**3.12**
*M. spicata*	2.25
*R. officinalis*	2.75

**Table 3:** Chemical composition of eight essential oils obtained by GC-MS.

Compound name	Area (%)							
	TV	OB	AHA	MS	MP	RO	EG	SA
*α*-Caryophyllene	—	—	—	—	—	—	—	3.74
*α*-Pinene	0.85	0.64	6.07	—	0.509	8.11	4.67	—
*α*-Terpinene	3.25	—	—	2.3	—	0.32	—	—
*α*-Thujone	—	—	9.26	—	—	—	—	—
*α*-Thujene	—	0.19	0.45	0.5	1.654	—	—	—
Eugenyl acetate	—	—	—	—	—	—	—	4.9
*α*-Terpineol	0.57	—	—	—	—	1.89	5.52	—
*β*-Pinene	1.63	0.83	1.09	2.4	0.896	1.48	2.07	—
*β*-Thujone	—	—	5.6	—	—	—	—	—
Borneol	0.65	—	—	—	—	6.18	—	—
Camphene	—	10.72	0.16	—	—	2.24	—	—
Camphor	**38.5**	0.3	**32.82**	—	—	**18.64**	—	—
Carvacrol	2.06	2.11	—	—	—	—	—	—
Carvone	—	0.23	—	**56.4**	—	—	—	—
Chrysanthenone	—	—	24.1	—	—	—	—	—
Crypton	—	—	—	—	—	—	3.13	—
Davanone	—	—	15.1	—	—	—	—	—
Eucalyptol (1.8 cineole)	5.45	10.5	—	2.98	0.19	**32.03**	**55.9**	—
Eugenol	—	2.4	—	—	—		—	**80.83**
*γ*-Terpinene	—	—	—	—	0.05	4.84	1.07	—
Globulol	—	—	—	—	—	—	12.99	—
Limonene	—	**13.2**	—	**16.2**	4.293	2.03	—	0.72
Linalool	1.79	**37.16**	—	7.98	—	1.47	0.24	—
Menthone	—	0.2	—	—	5.12	—	—	—
Methyl charviol	—	**14.3**	—	—	—	—	—	—
neo-menthol	—	—	—	—	**41.81**	—	—	—
Caryophyllene oxide	—	—	—	0.2	2.13	—	—	—
*p*-Cymene	1.19	3.1	—	—	0.72	0.31	—	—
Pulegone	—	—	—	—	**36.92**	—	—	—
Sabinene	0.33	0.5	—	0.6	0.642	0.28	—	0.11
Spathulenol	—	—	—	0.3	—	—	—	—
Thymol	**41.39**	2.1	—	—	—	0.98	—	—
Trycelene	—	—	0.14	—	—	—	—	—
Verbenene	0.13	—	—	—	—	3.6	—	—
Unidentified compounds	2.21	1.52	5.21	10.14	5.066	15.6	14.41	9.7
Total	97.79	98.48	94.79	89.86	94.934	84.4	85.59	90.3

TV: *T. vulgaris*; OB: *O. basilicum*; AHA: *A. herba-alba*; MS: *M. spicata*; MP: *M. pulegium*; RO: *R. officinalis*; EG: *E. globulus*; SA: *S. aromaticum*.

**Table 4:** Antibiotics sensitivity of *Escherichia coli* strains in different turkey organs.

Antibiotics	Streptomycin		Colistin		Gentamicin	
	ID (mm)	P	ID (mm)	P	ID (mm)	P
Thigh	12	R	15	R	20	S
Upper thigh	11	R	16	I	21	S
Breast	12	R	15	R	18	S
Skin	18	S	16	I	19	S
Throat	14	I	18	S	23	S
Wing	20	S	17	I	21	S
Liver	22	S	15	R	20	S

ID: inhibition zone diameter value; P: profile; R: resistant; I: intermediate; S: sensible.

**Table 5:** Inhibition zone diameter (mm) of the essential oils on *Escherichia coli* strain isolated from different organs of turkey.

Essential oil	Organs						
	Breast (mm)	Liver (mm)	Skin (mm)	Thigh (mm)	Throat (mm)	Upper thigh (mm)	Wing (mm)
*A. herba-alba*	16.33	20.18	15	18.25	15.58	16.91	15.66
*E. globulus*	8.1	10.2	8.16	9.33	9.58	9.5	8.58
*M. pulegium*	8.83	10.2	10.33	8.91	9	9.16	9.5
*M. spicata*	10.75	9.9	10.58	10.36	9.16	10.25	10.66
*O. basilicum*	17.83	14.3	20.66	14.08	15.83	12.25	15.5
*R. officinalis*	9.41	10.33	10.25	9.45	10.08	9.75	10.33
*S. aromaticum*	14	16.1	13.58	15.33	14.66	13.58	17.16
*T. vulgaris*	20.16	19.9	19.66	22.33	23.33	22.41	21.58

**Table 6:** Minimum inhibitory concentration (MIC), minimum bactericidal concentration (MBC), and the MIC/MBC ratio.

Essential oil	*E. coli*			
	MIC (v/v)	MBC (v/v)	MIC/MBC	Effect
*T. vulgaris*	1/3000	1/3000	1	Bactericidal
*A. herba-alba*	1/2000	1/2000	1	Bactericidal
*O. basilicum*	1/2000	1/2000	1	Bactericidal
*S. aromaticum*	1/1000	1/1000	1	Bactericidal
*R. officinalis*	1/250	1/250	1	Bactericidal
*M. spicata*	1/250	1/250	1	Bactericidal
*M. pulegium*	1/250	1/250	1	Bactericidal
*E. globulus*	1/100	1/100	1	Bactericidal

## Data Availability

All data supporting the findings are adequately included within the article.

## References

[1] FAO (Organisation des Nations unies pour lʼalimentation et lʼagriculture) (2012). Statistiques, disponible sur.

[2] Donoghue D. (2003). Antibiotic residues in poultry tissues and eggs: human health concerns?. *Poultry Science*.

[3] Ebani V. V., Nardoni S., Bertelloni F. (2016). Antibacterial and antifungal activity of essential oils against some pathogenic bacteria and yeasts shed from poultry. *Flavour and Fragrance Journal*.

[4] Bortolaia V., Espinosa-Gongora C., Guardabassi L. (2016). Human health risks associated with antimicrobial-resistant enterococci and *Staphylococcus aureus* on poultry meat. *Clinical Microbiology and Infection*.

[5] Ebani V. V., Nardoni S., Bertelloni F. (2019). In vitro antimicrobial activity of essential oils against *Salmonella enterica* serotypes Enteritidis and Typhimurium strains isolated from poultry. *Molecules*.

[6] Gonçalves-Tenório A., Silva B., Rodrigues V., Cadavez V., Gonzales-Barron U. (2018). Prevalence of pathogens in poultry meat: a meta-analysis of European published surveys. *Foods*.

[7] Chlebicz A., Śliżewska K. (2018). Campylobacteriosis, salmonellosis, yersiniosis, and listeriosis as zoonotic foodborne diseases: a review. *International Journal of Environmental Research and Public Health*.

[8] Dsani E., Afari E. A., Danso-Appiah A., Kenu E., Kaburi B. B., Egyir B. (2020). Antimicrobial resistance and molecular detection of extended spectrum *β*-lactamase producing *Escherichia coli* isolates from raw meat in Greater Accra region, Ghana. *BMC Microbiology*.

[9] Ebani V., Najar B., Bertelloni F., Pistelli L., Mancianti F., Nardoni S. (2018). Chemical composition and in vitro antimicrobial efficacy of sixteen essential oils against *Escherichia coli* and Aspergillus fumigatus isolated from poultry. *Veterinary Sciences*.

[10] Ngangom B. L., Tamunjoh S. S. A., Boyom F. F. (2019). Antibiotic residues in food animals: public health concern. *Acta Ecologica Sinica*.

[11] Chen J., Ying G.-G., Deng W.-J. (2019). Antibiotic residues in food: extraction, analysis, and human health concerns. *Journal of Agricultural and Food Chemistry*.

[12] Apata D. F. (2009). Antibiotic resistance in poultry. *International Journal of Poultry Science*.

[13] Gaggìa F., Mattarelli P., Biavati B. (2010). Probiotics and prebiotics in animal feeding for safe food production. *International Journal of Food Microbiology*.

[14] Vougat Ngom R. R., Foyet H. S., Garabed R., Zoli A. P. (2020). Human health risks related to penicillin G and oxytetracycline residues intake through beef consumption and consumer knowledge about Drug residues in Maroua, far North of Cameroon. *Frontiers in Veterinary Science*.

[15] Hashemi S. R., Davoodi H. (2012). Herbal plants as new immuno-stimulator in poultry industry: a review. *Asian Journal of Animal and Veterinary Advances*.

[16] Diaz Carrasco J. M., Redondo L. M., Redondo E. A., Dominguez J. E., Chacana A. P., Fernandez Miyakawa M. E. (2016). Use of plant extracts as an effective manner to control *Clostridium perfringens* induced necrotic enteritis in poultry. *BioMed Research International*.

[17] Abd El-Hack M. E., Alagawany M., Abdel-Moneim A.-M. E. (2020). Cinnamon (*Cinnamomum zeylanicum*) oil as a potential alternative to antibiotics in poultry. *Antibiotics*.

[18] Omidi M., Khosravinia H., Masouri B. (2020). Independent and combined effects of Satureja khuzistanica essential oils and dietary acetic acid on fatty acid profile in thigh meat in male broiler chicken. *Poultry Science*.

[19] Pham V. H., Kan L., Huang J. (2020). Dietary encapsulated essential oils and organic acids mixture improves gut health in broiler chickens challenged with necrotic enteritis. *Journal of Animal Science and Biotechnology*.

[20] Michalczyk M., Satora P., Banaś J., Fiutak G. (2020). Effect of adding essential oils of caraway and rosemary on volatile aroma compounds derived from stored vacuum packaged minced Turkey meat. *Annals of Animal Science*.

[21] Adaszyńska-Skwirzyńska M., Szczerbińska D. (2017). Use of essential oils in broiler chicken production–a review. *Annals of Animal Science*.

[22] Bakkali F., Averbeck S., Averbeck D., Idaomar M. (2008). Biological effects of essential oils - a review. *Food and Chemical Toxicology*.

[23] Idm’hand E., Msanda F., Cherifi K. (2020). Ethnopharmacological review of medicinal plants used to manage diabetes in Morocco. *Clinical Phytoscience*.

[24] Hayat J., Mustapha A., Abdelmajid M. (2020). Ethnobotanical survey of medicinal plants growing in the region of “oulad Daoud Zkhanine” (Nador province), in Northeastern Morocco. *Ethnobotany Research and Applications*.

[25] Hilali M. (2020). Aromatic and medicinal plants and their use of the Massmouda region Northern Morocco. *Plant Archives*.

[26] Jaber H., Ijoub R., Oubihi A. (2018). Antibioresistance of *Escherichia coli* strains isolated from Turkey meat marketed in Kenitra city (Morocco). *American Journal of Innovative Research and Applied Sciences*.

[27] Bennani H. (2000). Therapeutic use of antibiotics in poultry: current state in Morocco.

[28] CA-SFM (Comité De L’antibiogramme De La SFM (1993). Recommandation N3: antibiogramme par diffusion en milieu gélosé pour les bactéries aérobies á croissance rapide. *Bulletin-French Society for Microbiology*.

[29] Acar J., Bergogne-Berezin E., Chabber Y. (1993). Communiqué 1993 du Comité de l’Antibiogramme de la Socirté Française de Microbiologie. *Pathologie Biologie*.

[30] CA/SFM *Committee of the Susceptibility Bellowing to the French Society of Microbiology*.

[31] Candan F., Unlu M., Tepe B. (2003). Antioxidant and antimicrobial activity of the essential oil and methanol extracts of *Achillea millefolium* subsp. millefolium Afan. (Asteraceae). *Journal of Ethnopharmacology*.

[32] Oubihi A., Ouryemchi I., Nounah I. (2020). Chemical composition, antibacterial and antifungal activities of Thymus leptobotrys Murb essential oil. *Advances in Traditional Medicine*.

[33] El-Mahmood A. M., Doughari J. H. (2009). Bacteriological examination of some diluted disinfectants routinely used in the Specialist Hospital Yola, Nigeria. *African Journal of Pharmacy and Pharmacology*.

[34] Bourkhiss M., Hnach M., Bourkhiss B., Ouhssine & M., Chaouch A. (2007). Chemical composition and antimicrobial properties of essential oils extracted from the leaves of Tetraclinis articulata (Vahl) from Marocoo. *Journal of African Earth Sciences*.

[35] Oussalah M., Caillet S., Salmiéri S., Saucier L., Lacroix M. (2006). Antimicrobial effects of alginate-based film containing essential oils for the preservation of whole beef muscle. *Journal of Food Protection*.

[36] Remmal A., Tantaoui-Elaraki A., Bouchikhi T., Rhayour K., Ettayebi M. (1993). Improved method for determination of antimicrobial activity of Essential oils in agar medium. *Journal of Essential Oil Research*.

[37] Satrani B., Farah A., Fechtal M., Talbi M., Blaghen M. (2001). Composition chimique et activité antimicrobienne des huiles essentielles de Saturja calamintha et Saturja alpina du Maroc. *Annales des Falsifications et de l’expertise Chimique et Toxicologique*.

[38] Johari M. A., Khong H. Y. (2019). Total phenolic content and antioxidant and antibacterial activities of Pereskia bleo. *Advances in Pharmacological and Pharmaceutical Sciences*.

[39] Berche P., Gaillard J. L., Simonet M. (1991). *Les bactéries des infections humaines. Éditeur: Flammarion*.

[40] Rakholiya K. D., Kaneria M. J., Chanda S. V. (2015). In vitro assessment of novel antimicrobial from methanol extracts of matured seed karnel and leaf of *Mangifera indica* L. (Kesar Mango) for inhibition of *Pseudomonas* spp. and their synergistic potential. *American Journal of Drug Discovery and Development*.

[41] Abadi A. T. B., Rizvanov A. A., Haertlé T., Blatt N. L. (2019). World Health Organization report: current crisis of antibiotic resistance. *BioNanoScience*.

[42] Pieri A., Aschbacher R., Fasani G. (2020). Country income is only one of the tiles: the global journey of antimicrobial resistance among humans, animals, and environment. *Antibiotics*.

[43] Hafed Z., Benguedour R., Aboussaleh Y. (2015). Antibiotic resistance profile of *Escherichia coli* of avian origin: broiler case in the region of grand casablanca-Morocco. *American Journal of Innovative Research and Applied Sciences*.

[44] Filali E., Bell J., Elhouadfi M., Huggins M., Cook J. (1988). Antibiotic resistance of *Escherichia coli* strains isolated from chickens with colisepticaemia in Morocco. *Comparative Immunology, Microbiology and Infectious Diseases*.

[45] Bouchrif B., Paglietti B., Murgia M. (2009). Prevalence and antibiotic-resistance of Salmonella isolated from food in Morocco. *The Journal of Infection in Developing Countries*.

[46] Ennaji H., Timinouni M., Ennaji M. M., Hassar M., Cohen N. (2008). Characterization and antibiotic susceptibility of Listeria monocytogenes isolated from poultry and red meat in Morocco. *Infection and Drug Resistance*.

[47] Asmai R., Karraouan B., Es-Soucratti K. (2020). Prevalence and antibiotic resistance of Campylobacter coli isolated from broiler farms in the Marrakesh Safi region, Morocco. *Veterinary World*.

[48] Chaiba A., Filali F. (2018). Methicillin-resistant *Staphylococcus aureus* Nasal carriage in poultry farmers and poultry slaughterers in ouarzazate-Morocco. *Annual Research & Review in Biology*.

[49] Wink M. (2012). Secondary metabolites from plants inhibiting ABC transporters and reversing resistance of cancer cells and microbes to cytotoxic and antimicrobial agents. *Frontiers in Microbiology*.

[50] Pandey A. K., Kumar P., Singh P., Tripathi N. N., Bajpai V. K. (2017). Essential oils: sources of antimicrobials and food preservatives. *Frontiers in Microbiology*.

[51] Hmiri S., Rahouti M., Habib Z., Satrani B., Ghanmi M., El Ajjouri M. (2011). Evaluation du potentiel antifongique des huiles essentielles de mentha pulegium et d’eucalyptus camaldulensis dans la lutte biologique contre les champignons responsables de la détérioration des pommes en conservation. *Bulletin de la Société Royale des Sciences de Liége*.

[52] Farah A., Fechtal M., Chaouch A. (2002). Effet de l’hybridation interspécifique sur la teneur et la composition chimique des huiles essentielles d’eucalyptus cultivés au Maroc. *Biotechnology, Agronomy and Society and Environment*.

[53] Imelouane B., Amhamdi H., Wathelet J. P., Ankit M., Khedid K., El Bachiri A. (2009). Chemical composition of the Essential oil of thyme (*Thymus vulgaris*) from Eastern Morocco. *International Journal of Agriculture and Biology*.

[54] Akrout A. (2004). Essential oil study of some pastoral plants from Matmata (south Tunisia) [in French]. *Cahiers Options Méditerranéennes*.

[55] Cheurfa M., Allem R., Sebaihia M., Belhireche S. (2013). Effet de lʼhuile essentielle de Thymus vulgaris sur les bactéries pathogènes responsables de gastroentérites. *Phytothérapie*.

[56] Hanif M. A., Al-Maskari M. Y., Al-Maskari A., Al-Shukaili A., Al-Maskari A. Y., Al-Sabahi J. N. (2011). Essential oil composition, antimicrobial and antioxidant activities of unexplored Omani basil. *Journal of Medicinal Plants Research*.

[57] Kadri A., Zarai Z., Békir A., Gharsallah N., Damak M., Gdoura R. (2011). Chemical constituents and antioxidant activity of the essential oil from aerial parts of Artemisia herba-alba grown in Tunisian semi-arid region. *African Journal of Biotechnology*.

[58] Moemenbellah‐Fard M. D., Abdollahi A., Ghanbariasad A., Osanloo M. (2020). Antibacterial and leishmanicidal activities of *Syzygium aromaticum* essential oil versus its major ingredient, eugenol.. *Flavour and Fragrance Journal*.

[59] Paolini J., Ouariachi E., Bouyanzer A. (2010). Chemical variability of Artemisia herba-alba Asso essential oils from East Morocco. *Chemical Papers*.

[60] Lambert R. J. W., Skandamis P. N., Coote P. J., Nychas G.-J. E. (2001). A study of the minimum inhibitory concentration and mode of action of oregano Essential oil, thymol and carvacrol. *Journal of Applied Microbiology*.

[61] Al-Shuneigat J., Al-Sarayreh S., Al–Saraira Y., AlQudah M., Al-Tarawneh I., Al-Dalaen S. (2014). Chemical composition and antimicrobial activity of the essential oil of wild Thymus vulgaris grown in South Jordan. *IOSR Journal of Pharmacy and Biological Sciences*.

[62] Rota M. C., Herrera A., Martinez R. M., Sotomayor J. A., Jordan M. J. (2007). Antimicrobial activity and chemical composition of Thymus vulgaris, Thymus zygis and Thymus hyemalis Essential oils. *Food Control*.

[63] Ultee A., Slump R. A., Steging G., Smid E. J. (2000). Antimicrobial activity of carvacrol toward Bacillus cereus on rice. *Journal of Food Protection*.

[64] Zouari S., Zouari N., Fakhfakh N., Bougatef A., Ayadi M. A., Neffati M. (2010). Chemical composition and biological activities of a new essential oil chemotype of Tunisian Artemisia herbaalbaAsso. *Journal of Medicinal Plants Research*.

[65] Shirazi M. T., Gholami H., Kavoosi G., Rowshan V., Tafsiry A. (2014). Chemical composition, antioxidant, antimicrobial and cytotoxic activities of T agetes minuta and O cimum basilicum essential oils. *Food Science & Nutrition*.

[66] Oussalah M., Caillet S., Saucier L., Lacroix M. (2007). Inhibitory effects of selected plant essential oils on the growth of four pathogenic bacteria: *E. coli* O157:H7, Salmonella Typhimurium, *Staphylococcus aureus* and Listeria monocytogenes. *Food Control*.

[67] Bezza L., Mannarino A., Fattarsi K. (2010). Composition chimique de l’huile essentielle d’Artemisia herba-alba provenant de la région de Biskra (Algérie). *Phytothérapie*.

[68] Hussain A. I., Anwar F., Hussain Sherazi S. T., Przybylski R. (2008). Chemical composition, antioxidant and antimicrobial activities of basil (*Ocimum basilicum*) essential oils depends on seasonal variations. *Food Chemistry*.

[69] Moleyar V., Narasimham P. (1992). Antibacterial activity of essential oil components. *International Journal of Food Microbiology*.

[70] Ngom S., Diop M., Mbengue M., Faye F., Kornprobst J. M., Et Samb A. (2014). Composition chimique et propriétés antibactériennes des huiles essentielles d’Ocimum basilicum et *d’Hyptis suaveolens* (L.). *Poit récoltés dans la région de Dakar au Sénégal, Afrique Science*.

[71] Ghanmi M., Satrani B., Aafi A. (2010). Effet de la date de récolte sur le rendement, la composition chimique et la bioactivité des huiles essentielles de l’armoise blanche (Artemisia herba-alba) de la région de Guerçif (Maroc oriental). *Phytothérapie*.

[72] Leuschner R. G. K., Ielsch V. (2003). Antimicrobial effects of garlic, clove and red hot chilli onListeria monocytogenesin broth model systems and soft cheese. *International Journal of Food Sciences and Nutrition*.

[73] Silva L. F., Cardoso M. d. G., Batista L. R. (2015). Chemical characterization, antibacterial and antioxidant activities of essential oils of *Mentha viridis* L. and *Mentha pulegium* L. (L). *American Journal of Plant Sciences*.

[74] Gill A. O., Holley R. A. (2006). Disruption of *Escherichia coli*, Listeria monocytogenes and Lactobacillus sakei cellular membranes by plant oil aromatics. *International Journal of Food Microbiology*.

[75] O’Bryan C. A., Pendleton S. J., Crandall P. G., Ricke S. C. (2015). Potential of plant essential oils and their components in animal agriculture–in vitro studies on antibacterial mode of action. *Frontiers in Veterinary Science*.

[76] Seow Y. X., Yeo C. R., Chung H. L., Yuk H.-G. (2014). Plant essential oils as active antimicrobial agents. *Critical Reviews in Food Science and Nutrition*.

[77] Zhai H., Liu H., Wang S., Wu J., Kluenter A.-M. (2018). Potential of essential oils for poultry and pigs. *Animal Nutrition*.

[78] Simitzis P. E. (2017). Enrichment of animal diets with essential oils-a great perspective on improving animal performance and quality characteristics of the derived products. *Medicines*.

[79] Di Vito M., Cacaci M., Barbanti L. (2020). *Origanum vulgare* essential oil vs. a commercial mixture of essential oils: in vitro effectiveness on *Salmonella* spp. from poultry and swine intensive livestock. *Antibiotics*.

